# Circulating Tumor Cells: A Promising Biomarker in the Management of Nasopharyngeal Carcinoma

**DOI:** 10.3389/fonc.2021.724150

**Published:** 2021-10-27

**Authors:** Jiangtao Wu, Huijun Zhu, Feifei Gao, Rensheng Wang, Kai Hu

**Affiliations:** ^1^ Department of Radiation Oncology, The First Affiliated Hospital of Guangxi Medical University, Nanning, China; ^2^ Department of Radiation Oncology, The Second Affiliated Hospital of Guangxi Medical University, Nanning, China; ^3^ Department of Oncology, Shenzhen Yantian District People’s Hospital, Shenzhen, China

**Keywords:** circulating tumor cells (CTCs), biomarker (BM), clinical application, individualized treatment, nasopharyngeal carcinoma

## Abstract

Nasopharyngeal carcinoma (NPC) is a malignancy that arises from the mucosal epithelium of the nasopharynx, and its prognosis is relatively favorable. The 5-year overall survival rate in patients with locally advanced NPC currently exceeds 80%, but the development of individualized diagnosis and treatment at the molecular level is relatively lacking. Circulating tumor cells (CTCs) is the generic term for tumor cells that are present in the peripheral blood circulation. As a new biomarker with good clinical application prospects, the detection of CTCs has the advantages of being non-invasive, simple, and repeatable. By capturing and detecting CTCs in peripheral blood and monitoring the dynamic variation of its type and quantity, we can assess the biological characteristics of tumor in a timely manner and evaluate the therapeutic effect and prognosis of patients in advance, which will help to develop individualized treatments of tumors. The primary purposes of this review were the clinical application of CTCs in tumor stage determination, treatment efficacy evaluation, and prognosis prediction of NPC. In addition, we estimated the correlation between Epstein-Barr virus infection and CTCs and analyzed the difference in karyotypes and specific markers expressed on CTCs. We believe that our study will provide new insights and biomarkers for the individualized treatment of patients with NPC.

## Introduction

Nasopharyngeal carcinoma (NPC) is a malignancy with regional distribution characteristics that occurs mostly in southern China, Southeast Asia, and Northern Africa (1). Its pathogenesis is related to genetics, diet, environment, and Epstein-Barr virus (EBV) infection (2–4). Recently, with the development of radiotherapy technology and equipment, the 5-year overall survival (OS) of patients with locally advanced NPC has exceeded 80% (1, 5, 6), but the development of individualized diagnosis and treatment at the molecular level is relatively lacking. Therefore, identification of accurate and effective biomarkers is key to further improving the therapeutic effect and realizing individualized treatments for patients with NPC.

**Figure 1:**
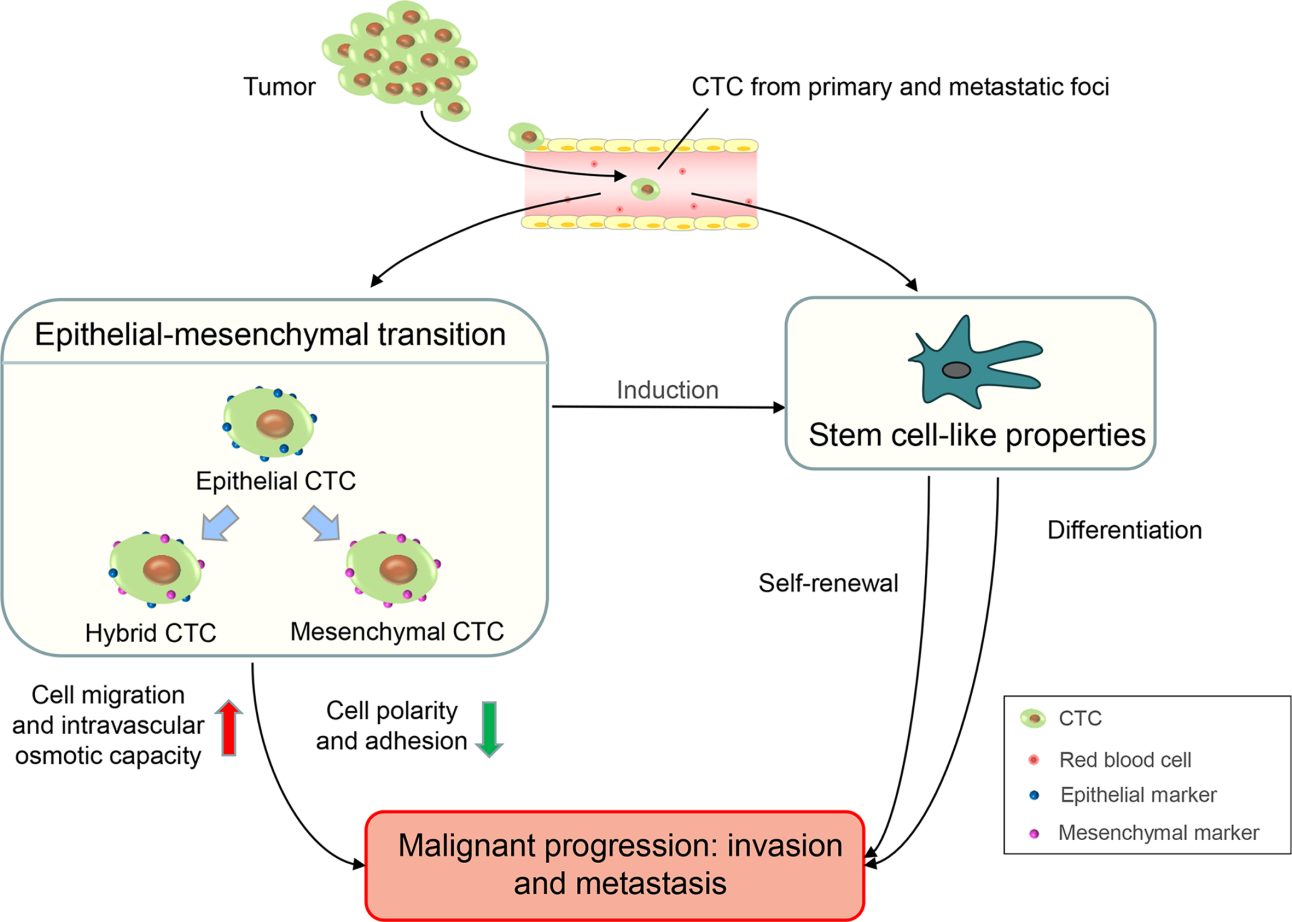
The biological properties of CTCs and their effects on tumors invasion and metastasis.

Circulating tumor cells (CTCs) are tumor cells that are shed from primary or metastatic foci and are present in the peripheral blood (7). Many specific protein markers occur on the surface of CTCs. These differentially expressed protein markers are the biological basis for the separation and detection of CTCs in peripheral blood. Epithelial cell adhesion molecules (EpCAMs) and cytokeratins (CKs) are overexpressed in CTCs, and these are specific epithelial markers commonly used to detect CTCs (8). The lack of CD45 expression on the surface and the presence of the nucleus are also characteristic of CTCs. Therefore, CTCs have a characteristic immunophenotype of ‘EpCAM+/CK+/CD45-’ which exists in the peripheral blood circulation of patients with cancer but not in healthy individuals or patients with non-malignant diseases (9). In recent years, the epithelial-mesenchymal transition (EMT) and stem cell-like properties of CTCs have been studied extensively (10). Tumor cells can obtain mesenchymal characterization through EMT such as the loss of cell polarity and adhesion, enhanced cell migration, and intravascular osmotic ability, which leads to the progression and metastasis of malignancy (11, 12). According to EMT markers, such as epithelial markers (EpCAMs or CKs) and mesenchymal markers (Vimentin or Twist), CTCs can be divided into three subgroups: epithelial CTCs (only epithelial markers), mesenchymal CTCs (only mesenchymal markers), and hybrid CTCs (both epithelial and mesenchymal markers). Among them, mesenchymal and hybrid CTCs are more aggressive owing to the acquisition of mesenchymal phenotypic characteristics and are often associated with disease progression (13, 14). Evidence shows that cancer cells contain abundant cell types and are highly heterogeneous. Cancer stem cells (CSCs) are a subgroup of cancer cells with stem cell properties. CSCs have a strong potential for self-renewal and differentiation which frequently lead to tumor progression, therapy resistance, and distant metastasis (15, 16). The presence of stem cell characteristics has been observed in CTCs (17). In *ex-vivo* cultures of CTCs from patients with colorectal cancer, Grillet et al. (18) reported that CTC lines exhibit hallmarks of CSCs and possess strong metastatic potential. Similar to EMT and stem cell-like properties, which lead to tumor progressions such as invasion and metastasis, CTCs have an important application value in clinical diagnosis, therapeutic effect evaluation, and prognosis prediction of malignancies (**Figure 1**) .

**Figure 2:**
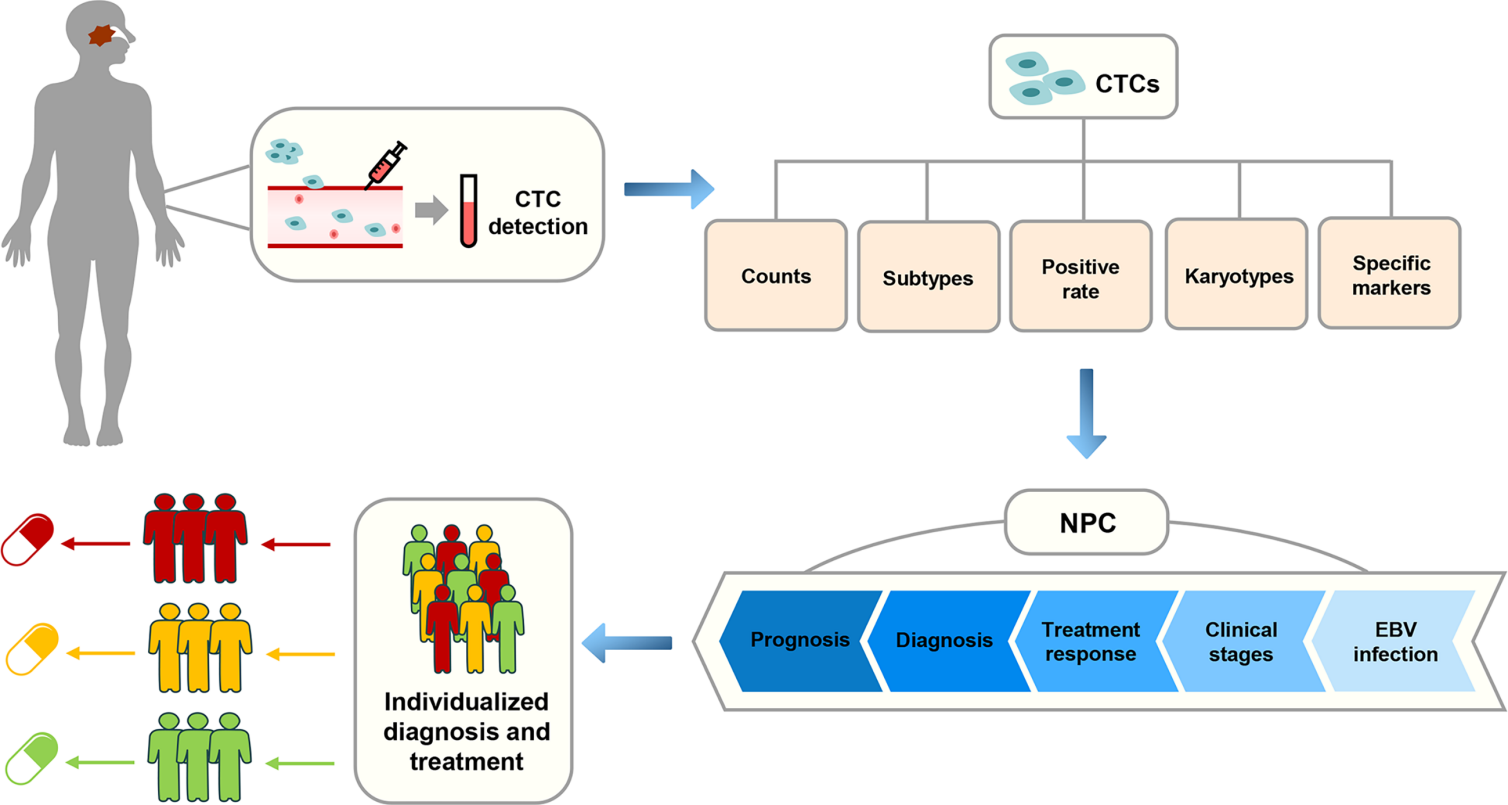
The correlation between the application of CTCs and the individualized diagnosis and treatment of NPC in clinical practice.

As one of the main detection targets of liquid biopsy, CTCs have been widely used in many studies, including those involving lung cancer, gastric cancer, breast cancer, and colorectal cancer (19–22). In the past few years, many scholars have conducted extensive research on the application of CTCs in NPC. In this review, we assessed the progress of the clinical application of CTCs and expounded the research status and challenges of CTCs in the diagnosis and treatment of NPC to provide new insights and biomarkers for the realization of individualized diagnosis and treatment of patients with NPC (**Figure 2**). A summary of the current research on CTCs in NPC is shown in **Table 1**.

**Table 1:** Summary of studies on CTCs in nasopharyngeal carcinoma.

Author	Years	Patients	CTCs detection technology	Detection markers	Specific markers	Sampling time	Clinical stages	Treatment response	Prognosis	Main conclusions
Xie et al. (23)	2019	50	The CanPatrol™ system	CD45, EpCAM, CK8/18/19, Vimentin, Twist	COX-2	Before and after TM	√	√	–	Decreased mesenchymal CTCs and that express COX-2 indicated a favorable therapeutic effect in NPC patients.
Zhang et al. (24)	2018	50	SE-iFISH	CD45, EpCAM, CEP8,	–	Before and after TM	√	√	–	Real-time monitoring the changes in CTCs number may predict the chemotherapy efficacy during treatment. Triploid and multiploid CTCs may related to chemo-resistance.
Wen et al. (25)	2019	60	The CanPatrol™ system	CD45, CK18, Twist	N-cadherin	Before and after TM	√	√	√	The increased number of total and mesenchymal CTCs after treatment indicated poor therapeutic effects. Mesenchymal CTCs may serve as a predictor of PFS.
Zeng et al. (26)	2019	60	The Cyttel™ CTC detection	CD45, CEP8	–	Before and after TM	√	√	√	Patients with CTCs positive had a later N stage and a shorter OS.
Liu et al. (27)	2020	135	The Cyttel™ CTC detection	CD45, CEP8	–	Before and after TM	√	–	√	After treatment, CTCs-positive patients’ PFS and OS were significantly lower than those in CTCs-negative patients.
Si et al. (28)	2016	81	The CanPatrol™ system	CD45, CK19, Twist	MMP9	Before and after TM	√	√	–	Decreased CTCs in NPC patients indicated a favourable therapeutic effect. Hybrid and mesenchymal CTCs were significantly correlated with metastasis. MMP9 existed in CTCs and expressed most in mesenchymal CTCs.
Qian et al. (29)	2019	50	The Cyttel™ CTC detection	CD45, CEP8	–	Before and after TM	√	√	–	The decrease in CTCs number during chemotherapy and radiotherapy suggested a favourable therapeutic effect.
Li et al. (30)	2016	38	The Cyttel™ CTC detection	CD45, CEP8	–	Before and after TM	√	√	–	There was no statistically significant correlation between CTC number or positivity and clinical parameters like TNM stages, but CTCs number was remarkably decreased after radiotherapy.
Ou et al. (31)	2019	370	The CellSearch^®^ system	CD45, EpCAM, CK8/18/19	–	Before and after TM	√	–	√	The more CTCs count, the worse prognosis in patients with advanced NPC.
Wu et al. (32)	2017	67	Antibody labeling and flow cytometry	CD45, EpCAM, CK	–	Before TM	√	–	√	Positive CTCs in peripheral blood was associated with N staging and indicated a poor prognosis in NPC patients.
Cai et al. (33)	2011	76	IME-FICC	EpCAM, CK8/18	–	Before TM	–	–	√	CTCs’ positive rate was associated with recurrence-free survival and overall survival.
Li et al. (34)	2018	131	The CanPatrol™ system	CD45, EpCAM, CK8/18/19, Vimentin, Twist	COX-2	Before and after TM	√	√	√	The COX-2 expression on CTCs after treatment was associated with poor therapeutic effect and the high risk of recurrence and metastasis.
Yu et al. (35)	2020	179	The CanPatrol™ system	CD45, EpCAM, CK8/18/19, Vimentin, Twist	FN1	Before and after TM	√	–	√	NPC patients with high expression of FN 1 on CTCs had a poor prognosis.
Fu et al. (36)	2017	33	qRT-PCR	–	hTERT mRNA	Before and after TM	√	√	–	Radiochemotherapy can effectively reduce the hTERT mRNA levels in peripheral blood and CTCs of NPC patients. Combined detection of hTERT mRNA levels and CTCs in peripheral blood can become a new biomarker for therapeutic effect and prognosis of NPC.
He et al. (37)	2017	33	ISET and IHC staining	CK5/6, P63	–	Before and after TM	√	–	–	There was a positive correlation between CTCs and EBV activation in NPC patients.
Wu et al. (38)	2015	63	The Cyttel™ CTC detection	CD45, CEP8	–	Before TM	√	–	–	CTCs were closely related to NPC metastasis and EBV-DNA.
Mao et al. (39)	2019	49	The CanPatrol™ system	CD45, EpCAM, CK8/18/19, Vimentin, Twist	–	Before TM	√	–	–	CTCs were correlated with systemic metastasis and EBV DNA in NPC patients.
Vo et al. (40)	2016	46	Microsieve technology	CD45, EpCAM, CK	–	Before and after TM	√	√	√	EBV cfDNA outperforms CTC enumeration in correlation with clinical outcomes of NPC patients undergoing treatment.
You et al. (41)	2019	148	The CellSearch^®^ system	CD45, EpCAM, CK8/18/19	–	Before and after TM	√	√	√	Compared to EBV DNA, CTCs showed superior specificity but inferior sensitivity in the detection of metastatic NPC. In metastatic NPC patients, the number of CTCs and EBV DNA before, after and during first-line chemotherapy was strong predictive markers for PFS and OS, respectively.

**The CanPatrol™ system**, includes CTC-enrichment technique and in situ hybridization; **The Cyttel™ CTC detection**, includes negative enrichment and immunoﬂuorescence in situ hybridization; **SE-iFISH**, subtraction enrichment and immunostaining fluorescence in situ hybridization; **IME-FICC**, immunomagnetic enrichment of tumor cells in combination with fluorescent immunocytochemistry; **qRT-PCR**, real-time quantitative reverse transcription polymerase chain reaction; **ISET**, isolation by size of epithelial tumor; IHC, Immunohistochemical; EpCAM, epithelial cell adhesion molecules; CK, cytokeratin; CEP8, centromere probe 8; TM, treatment; “√” indicates that the literature involves relevant content.

## Clinical Significance of CTCs in NPC Staging

The Tumor-Node-Metastasis (TNM) staging system of the American Joint Committee on Cancer/Union for International Cancer Control is the most widely used staging system worldwide (42). However, with the development of precision medicine, the current staging system which is based on the anatomical structure can no longer meet the needs of individualized treatment. Therefore, many studies have begun to introduce new biomarkers into the staging system for the exploration of individualized treatment (43–45). However, there is no consensus on the relationship between the clinical stages of patients with NPC and the positive rate or number of CTCs.

By detecting CTCs in 10 healthy volunteers and 50 patients with NPC, Xie et al. (23) found that 48 (96%) patients with NPC were CTC-positive, but CTCs were not detected in the healthy volunteers. Meanwhile, CTC positivity was related to different NPC clinical stages, and in all the clinical stages, patients with stage IV had the highest mesenchymal CTC positivity. In a retrospective study including 50 patients with NPC, Zhang et al. (24) found that a significant correlation did not exist between the detection rate of CTCs and clinical stage. However, the number of CTCs increased consistently with clinical stage regardless of whether patients were newly diagnosed or had loco-regional recurrence or distant metastasis. In another study of 60 patients with NPC, Wen et al. (25) demonstrated that the positive rates of CTCs in stage II, III, and IV disease were 100%, 88.9%, and 83.8%, respectively, and there was no significant association between the positive rate or number of CTCs and TNM stage. However, Zeng et al. (26) found that the positive rate of CTCs was correlated with the N stage of NPC, and CTC-positive patients had a later N stage. Similarly, Liu et al. (27) reported that the more advanced the clinical stage of patients with NPC, the higher the positive rate of CTCs. Si et al. (28) also showed that there was a significant positive correlation between mesenchymal CTCs and the metastasis of NPC, especially lymph node metastasis. In summary, as one of the main detection targets of liquid biopsy, the positive rate and number of CTCs, along with their relationship with NPC stage, remain controversial and need to be further validated through large-scale prospective data.

## CTCs in the Early Evaluation of Treatment Efficacy for NPC

Patients with NPC generally choose to evaluate treatment efficacy at 3 months after completing radiotherapy because of the presence of inflammation and oedema of local soft tissues and the sublethal and potentially lethal damage to tumor cells caused by irradiation. However, for some patients who are resistant to radiotherapy, a 3-month waiting time may delay adjuvant therapy. Therefore, researchers have begun to search for more accurate and earlier indicators of treatment efficacy to adjust therapeutic regimens for treatment-resistant NPC patients as early as possible. At present, CTCs are increasingly applied in this field, in addition to the detection of EBV DNA.

A prospective study of locally advanced NPC by Qian et al. (29) found that 27 CTC-positive patients had a significant decrease in CTC count after radiotherapy and chemotherapy. Moreover, compared with patients presenting a partial response (PR), those presenting a complete response (CR) had a significant reduction in CTC count. After comparing the changes in the number of CTCs before and after treatment, Wen et al. (25) found that the number in patients with NPC who achieved CR or PR after treatment often decreased or remained unchanged, while the number in patients with progressive disease (PD) often increased. They also suggested that a post-treatment increase in the number of total and mesenchymal CTCs was often indicative of poor treatment outcomes. Li et al. (30) conducted a retrospective study on 38 patients with NPC. After analyzing the CTCs counts of patients before, 1 week after, and 1 month after radiotherapy, they found that the number of CTCs was significantly reduced at 1 month after radiotherapy. Another study (23) showed that the difference values (i.e., the difference value between the CTC counts before and after treatment) of total CTCs, epithelial CTCs, mesenchymal CTCs, and hybrid CTCs were all greater in NPC patients with CR than in those with PR, stable disease (SD), or PD. Similarly, Zhang et al. (24) found that the CTC count decreased after treatment in patients with PR, while it increased in those with PD/SD. The aforementioned indicates that the change in CTC counts was related to the therapeutic effect, which could not only provide an important reference for early evaluation of treatment efficacy but also contribute to timely adjuvant therapy for patients with NPC.

## Clinical Significance of CTCs in the Prognosis of NPC

CTCs are malignant cells that detach from primary or metastatic foci and enter blood vessels, and they can spread to distant organs through peripheral blood circulation and develop into possible metastatic foci (7, 46). Their number is recognized to be associated with tumor burden. As a target of real-time liquid biopsy, CTCs have also increasingly been identified as a potential biomarker to assess the biological characteristics of tumors and predict the prognosis of patients. Accordingly, they have attracted attention in clinical practice.

In a retrospective study involving 370 patients with NPC, Ou et al. (31) confirmed that CTC count was an independent risk factor for poor outcomes by using sex, age, EBV DNA, and CTC count as variables in the Cox regression model. In particular, for patients with advanced NPC (70 cases of stage III and 288 cases of stage IV), the Kaplan-Meier survival analysis revealed that a higher CTC count was associated with a worse prognosis. Wen et al. (25) reported that mesenchymal CTCs could be used as a predictor of progression-free survival (PFS) in patients with NPC. Among 36 patients treated with cisplatin and paclitaxel, mesenchymal CTCs were significantly associated with PFS following therapy, and the PFS of patients who were mesenchymal CTC-negative was notably better than that of mesenchymal CTC-positive patients. Wu et al. (32) analyzed the survival of 67 patients with NPC and showed that the 3-year OS and PFS rates were significantly better in the CTC-negative group (100% and 85.7%, respectively) than in the CTC-positive group (92.9% and 73.7%, respectively). Cai et al. (33) indicated that CTC-positive NPC patients had shorter OS and lower relapse-free survival rates than CTC-negative NPC patients. This result was consistent with the findings reported by Liu et al. (27), in which patients with NPC with elevated CTC counts had significantly lower PFS than those with decreased or unchanged CTC counts after treatment. Si et al. (28) also showed that decreased CTC counts after treatment suggested a favorable therapeutic effect in patients with NPC. Therefore, CTC count has the potential to predict the prognosis of patients with NPC, and CTC positivity is often associated with poor prognosis.

## Clinical Application of CTC Karyotyping in NPC

Nearly all mammals are diploid, whereas most human solid tumors are aneuploid and range from hypodiploid to hypertetraploid. Abnormal chromosomes are considered to be a vital factor for tumorigenesis (47). With the development of immunofluorescence *in situ* hybridization and chromosome probes, karyotypic analysis of CTCs can now be performed conveniently (48). Karyotypic analysis of chromosome 8 ploidy is the most well-developed and widely used method (49, 50).

CTC karyotypic analysis upon ploidy of chromosome 8 in 50 patients with NPC by Zhang et al. (24) revealed that the ratio of triploid CTCs was the highest compared with other karyotypes in newly diagnosed patients (M0), while the number of polyploid CTCs was the largest in those with recurrence or distant metastasis. However, there was no significant correlation between CTC count and different karyotypes and TNM stage. Their study also indicated that the ploidy of chromosome 8 in CTCs was associated with the efficacy of chemotherapy. In comparison, the tetraploid CTC count was decreased to a greater extent than both triploid and multiploidy CTC counts after chemotherapy with gemcitabine plus cisplatin. Specifically, the proportion of tetraploid CTCs was significantly decreased in all patients. Nevertheless, the ratio of triploid and multiploid CTCs all showed only a slight decline in PR patients after treatment. Moreover, for PD/SD patients, the frequency of triploid CTCs was increased from 66.7% to 100%, and multiploid CTCs remained the same after treatment. Similarly, a study performed by Qian et al. (29), showed that, after chemoradiotherapy, different karyotypes of CTCs were all significantly decreased in patients with NPC with CR, whereas statistical significance was not reached in those without CR. Their results also indicated that fewer tetraploid CTCs were correlated with CR in patients with locally advanced NPC. Notably, these studies suggested that CTC karyotyping is related to treatment response and can be used as a new biomarker for the prediction of chemoradiotherapy sensitivity and treatment efficacy.

## Research Progress of Specific Markers on CTCs in NPC

CTCs are considered to be ‘miniature’ versions of primary tumors which exist in peripheral blood circulation, and the expression of phenotypic and genetic markers on CTCs can reflect the biological characteristics of primary tumors. At present, the detection of specific markers on CTCs is an important breakthrough in studying the biological behavior of primary tumors and guiding clinical diagnosis and treatment.

Cyclooxygenase-2 (COX-2) is an inducible enzyme involved in cell proliferation and tumor angiogenesis and plays a pivotal role in tumor genesis and development (51). A prospective study by Li et al. (34) stated that COX-2 can be expressed on the CTCs of patients with NPC before and after treatment. COX-2 expression on CTCs after treatment was markedly associated with poor treatment response and a high risk of tumor recurrence and metastasis and can thus be used as an independent prognostic indicator for poorer OS and PFS. Xie et al. (23) found that the positivity of COX-2 on CTCs was higher in patients with NPC with stage IV than in those with stage II and III. Moreover, the decline of mesenchymal CTCs which express COX-2 was related to a favorable therapeutic effect for NPC.

Matrix metalloproteinase 9 (MMP-9) is a zinc-containing endopeptidase that participates in the degradation of the extracellular matrix and vascular reconstruction and is associated with tumor invasiveness and progression (52–54). Si et al. (28) confirmed that MMP-9 could be expressed in different types of CTCs in NPC and was mostly expressed in mesenchymal CTCs. However, there were no correlations between MMP-9 and clinical stage, EBV, and other characteristics like smoking in patients with NPC. Interestingly, Liu et al. (55) found that the overexpression of MMP-9 was associated with poor prognosis in patients with NPC.

N-cadherin is a Ca2+-dependent cellular adhesion molecule that is closely related to EMT, and its expression plays a prominent role in tumor invasion and metastasis (56–58). Wen et al. (25) indicated that N-cadherin can be expressed in epithelial, mesenchymal, and hybrid CTCs, but its overexpression was rare in all three types. The relationship between N-cadherin expression on CTCs and clinical parameters of NPC should be studied further.

Fibronectin 1 (FN1) is an important extracellular matrix molecule that plays an important role in cell adhesion, migration, and carcinogenesis (59). Yu et al. (35) found that FN1 expression on CTCs was correlated with the clinical stages of NPC, and patients with stage IV had the highest number of CTCs expressing FN1. Furthermore, it has been suggested that FN1 overexpression on CTCs is often associated with poor survival in patients with NPC.

Hence, combining the detection of specific surface markers on CTCs with clinical characteristics may be a new direction to guide clinical decision-making and realize individualized treatment for malignancy in the future. Besides the specific markers on CTCs, the transcriptome of CTCs has also been studied. Fu et al. (36) found that increased human telomerase reverse transcription (hTERT) mRNA levels in CTCs and peripheral blood of patients with NPC were closely related to the later clinical stage. Moreover, chemoradiotherapy was an effective method to reduce hTERT mRNA levels in CTCs and peripheral blood. Nevertheless, studies focusing on the transcriptome of CTCs are still limited.

## Relationship Between CTCs and EBV DNA in NPC

NPC is invariably associated with EBV infection (60). Circulating cell-free EBV DNA consisting of short DNA fragments released by NPC cells can be detected using ultrasensitive polymerase chain reaction-based assays (3, 61). Therefore, the detection of this biomarker presents an important clinical application in population screening, risk stratification, efficacy monitoring, and prognosis prediction in patients with NPC (62, 63). Recently, some studies have found that the coding products of EBV can promote the generation of CTCs (64), and the combined application of EBV and CTCs has become a hotspot in the diagnosis and treatment of NPC.

He et al. (37) found that serum EBV capsid antigen IgA (EBV VCA-IgA) levels in CTC-positive NPC patients were higher than those in CTC-negative NPC patients, and CTC enumeration was positively correlated with the EBV DNA load and EBV VCA-IgA levels. A study conducted by Wu et al. (38) involving 63 patients with NPC revealed that the positive rate of CTCs was notably correlated with the EBV DNA status, and it was significantly lower in EBV DNA-negative patients than in EBV DNA-positive patients. Moreover, Mao et al. (39) came to similar conclusions. Xie et al. (23) reported that EBV DNA-positive NPC patients had both a higher proportion of mesenchymal CTCs and expression of COX-2 than EBV DNA-negative patients. Thus, CTCs and COX-2 expression are associated with EBV DNA in patients with NPC; however, the mechanisms remain unclear. Interestingly, Vo et al. (40) found that there was no significant correlation between CTC counts and EBV DNA levels, and this may be due to the different CTC detection techniques which need to be studied further.

A prospective cohort study of metastatic NPC performed by You et al. (41) found that the variations between CTC counts and EBV DNA copies at baseline and after first-line chemotherapy were important independent prognostic factors for PFS and OS, respectively. Furthermore, they suggested that CTC count had superior specificity but inferior sensitivity when compared with plasma EBV DNA copies in the diagnosis of distant metastasis of NPC. Therefore, the combination of CTCs and EBV DNA was a stronger predictive marker than a single indicator, and when utilized in conjunction with imaging, it could provide additional information for individual diagnosis and treatment of NPC.

## Circulating Tumor Cell Microemboli in NPC

The CTC cluster is composed of two or more tumor cells, also known as circulating tumor cell microemboli (CTM), which has 23–50 times the metastatic potential compared with a single CTC for cancer patients (65). Although CTM can be detected in the peripheral blood of patients with cancer, the number is relatively low. Among the known studies about CTCs in NPC, CTM was only detected in the study of He et al. (37) and Zhang et al. (24) with a detection rate of 6% (2/33) and 12% (6/50), respectively. However, the clinical significance of CTM was not clarified for patients with NPC in the two studies. Thus, the mechanism of CTM metastasis and its clinical value for NPC still need to be explored further.

## Conclusion

With the development of liquid biopsy techniques, CTCs, as a potential new biomarker, have an important clinical application value in determining the clinical stage, evaluating treatment efficacy, and predicting the prognosis of NPC. Meanwhile, different karyotypes and specific markers expressed on CTCs in patients with NPC can provide further information regarding chemoradiotherapy sensitivity and treatment response. Thus, CTCs would be a good addition to the existing TNM staging system based on the anatomical structure; furthermore, it could be advantageous in providing better individualized treatments for patients with NPC.

However, the current clinical application of CTCs in NPC still presents many limitations. Most reported studies are retrospective analyses with a small samples size, and the technical methods for CTC detection vary, leading to inconsistent results. Meanwhile, there are many challenges in current research, such as the marked inter- and intra-patient heterogeneity of the phenotypic and genetic markers on CTCs. Therefore, considering a unified and standardized detection technology, searching for new CTC-specific markers, and performing prospective large-scale clinical research are promising ways for CTC detection to be widely applied in clinical practice and to further achieve individualized treatments for patients with NPC in the future.

## Author Contributions

RW and KH designed and revised the manuscript and participated in the literature analysis. JW and KH searched the literature, wrote the manuscript, and created the figures and tables. JW, HZ, and FG coordinated, edited, and finalized the drafting of the manuscript. All authors contributed to the article and approved the submitted version.

## Funding

This work was supported by grants from the National Natural Science Foundation of China (No. 82060019), the Natural Science Foundation of Guangxi (2018JJA140869), and the Guangxi Medical and Health Appropriate Technology Development and Application Project (S2018097).

## Conflict of Interest

The authors declare that the research was conducted in the absence of any commercial or financial relationships that could be construed as a potential conflict of interest.

## Publisher’s Note

All claims expressed in this article are solely those of the authors and do not necessarily represent those of their affiliated organizations, or those of the publisher, the editors and the reviewers. Any product that may be evaluated in this article, or claim that may be made by its manufacturer, is not guaranteed or endorsed by the publisher.
